# The Tolerogenic Peptide, hCDR1, Down-Regulates the Expression of Interferon-α in Murine and Human Systemic Lupus Erythematosus

**DOI:** 10.1371/journal.pone.0060394

**Published:** 2013-03-28

**Authors:** Zev Sthoeger, Heidy Zinger, Amir Sharabi, Ilan Asher, Edna Mozes

**Affiliations:** 1 Department of Immunology, The Weizmann Institute of Science, Rehovot, Israel; 2 Department of Internal Medicine B and Clinical Immunology, Kaplan Medical Center, Rehovot, Israel; Johannes Gutenberg University of Mainz, Germany

## Abstract

**Background:**

The tolerogenic peptide, hCDR1, ameliorated manifestations of systemic lupus erythematosus (SLE) via the immunomodulation of pro-inflammatory and immunosuppressive cytokines and the induction of regulatory T cells. Because type I interferon (IFN-α) has been implicated to play a role in SLE pathogenesis, we investigated the effects of hCDR1 on IFN-α in a murine model of SLE and in human lupus.

**Methodology/Principal Findings:**

(NZBxNZW)F1 mice with established SLE were treated with hCDR1 (10 weekly injections). Splenocytes were obtained for gene expression studies by real-time RT-PCR. hCDR1 down-regulated significantly IFN-α gene expression (73% inhibition compared to vehicle treated mice, p = 0.002) in association with diminished clinical manifestations. Further, hCDR1 reduced, in vitro, IFN-α gene expression in peripheral blood mononuclear cells (PBMC) of 10 lupus patients (74% inhibition compared to medium, p = 0.002) but had no significant effects on the expression levels of IFN-α in PBMC of primary anti-phospholipid syndrome patients or of healthy controls. Lupus patients were treated for 24 weeks with hCDR1 (5) or placebo (4) by weekly subcutaneous injections. Blood samples collected, before and after treatment, were frozen until mRNA isolation. A significant reduction in IFN-α was determined in hCDR1 treated patients (64.4% inhibition compared to pretreatment expression levels, p = 0.015). No inhibition was observed in the placebo treated patients. In agreement, treatment with hCDR1 resulted in a significant decrease of disease activity. IFN-α appears to play a role in the mechanism of action of hCDR1 since recombinant IFN-α diminished the immunomodulating effects of hCDR1 on IL-1β, TGFβ and FoxP3 gene expression.

**Conclusions/Significance:**

We reported previously that hCDR1 affected various cell types and immune pathways in correlation to disease amelioration. The present studies demonstrate that hCDR1 is also capable of down-regulating significantly (and specifically to lupus) IFN-α gene expression. Thus, hCDR1 has a potential role as a novel, disease specific treatment for lupus.

## Introduction

Systemic lupus erythematosus (SLE) is an autoimmune disorder characterized by the production of autoantibodies and impaired function of B and T cells accompanied by systemic clinical manifestations [1]. Various cytokines [2], [3], factors affecting B cell activation and survival [4], apoptosis [5], [6] and dysfunction of regulatory T-cells [7], [8] were shown to be involved in the pathogenesis of murine and human SLE. Our laboratory designed a peptide, designated hCDR1 [9], that is based on the sequence of the complementarity determining region 1 (CDR1) of a human anti-DNA monoclonal antibody that bears a major idiotype (Id), namely the 16/6 Id [10]. hCDR1 was shown to ameliorate the serological and clinical manifestations of experimental SLE in mice with either induced (BALB/c) or spontaneous (NZBxNZW)F1 lupus [11]. The beneficial effects of hCDR1 were associated with a reduced production and expression of inflammatory cytokines (e.g. IL-1β, IFN-γ, TNF-α,) [11], [12] and up regulation of the immunosuppressive cytokine TGFβ [11], [13]. hCDR1 was shown to inhibit T cell receptor signaling following its binding to class II major histocompatibility complex (MHC) [14]. The induction of CD4 and CD8 regulatory T cells play a key role in the mechanism of action of hCDR1 [15]–[17]. Further, hCDR1 was shown to diminish T cell apoptosis [18], [19]. Treatment with hCDR1 affected the B cell compartment as well. Thus, it down regulated the rate of maturation, differentiation and survival of B cells by reducing the levels of B cell activating factor (BAFF, BLyS) [20] as well as of molecules of the CD74/MIF pathway on B cells [21]. In addition, hCDR1 was shown to induce dendritic cells with immature phenotype and suppressed function that down regulate autoreactive T cells [22].

We have further demonstrated similar immunomodulatory effects of hCDR1 on peripheral blood mononuclear cells (PBMC) obtained from lupus patients. Thus, incubation of PBMC of lupus patients (but not of healthy volunteers) with hCDR1 resulted in a significant down regulation of gene expression of pro-inflammatory cytokines, apoptotic factors, and BLyS and up regulation of gene expression of immunosuppressive factors (Foxj1, Foxo3a, TGFβ, Foxp3) [23]. In addition, hCDR1 increased the number as well as the function of CD4+CD25+Foxp3+regulatory T-cells in PBMC of lupus patients [23]. Further, we reported the beneficial effects of in vivo treatment with hCDR1 in five lupus patients with mild to moderate disease [24]. In agreement with its clinical beneficial effects, hCDR1 was shown to immunomodulate in vivo the expression of genes that play a role in SLE thus restoring the global immune dysregulation in those patients [24].

Type I interferons, mainly interferon (IFN)-α, were suggested to play a major role in the pathogenesis of murine and human SLE [25]. Thus, elevated levels of IFN-α were demonstrated in sera of SLE afflicted mice as well as in sera of lupus patients [26], [27], and IFN-α levels were reported to correlate with disease activity [28]. Similarly, high levels of IFN-α inducible gene expression (“IFN-signature”) were shown in blood cells of lupus patients [29]. Moreover, type I IFN receptor deficiency was reported to reduce significantly lupus like disease in a mouse model [30] and IFN-α neutralizing antibodies were shown to prevent the clinical manifestations in a lupus flare murine model [31]. Hence, IFN-α has been considered recently as a therapeutic target for the treatment of human SLE.

Since we have demonstrated that hCDR1 was capable of restoring the cytokine dysregulation observed in SLE and of down regulating the maturation and function of dendritic cells (that are activated by IFN-α and are a source for IFN-α production as well) we investigated, in the present studies, the effects of hCDR1 on IFN-α in a murine model of SLE and in human lupus.

## Results

### Treatment of SLE afflicted (NZBxNZW)F1 mice with hCDR1 down regulated gene expression of IFN-α

Eight month old (NZBxNZW)F1 female mice were treated with 10 subcutaneously weekly injections of hCDR1 (50 µg/mouse), control (scrambled) peptide (50 µg/mouse) or the vehicle alone (10–12 mice per group in 4 independent experiments). Mice were bled periodically for the determination of sera anti-dsDNA autoantibody and IFN-α levels and were tested for proteinuria levels. All mice were sacrificed at the end of treatment, their kidneys were evaluated for immune complex deposits and spleen derived lymphocytes were used for mRNA preparation.

**Figure 1:**
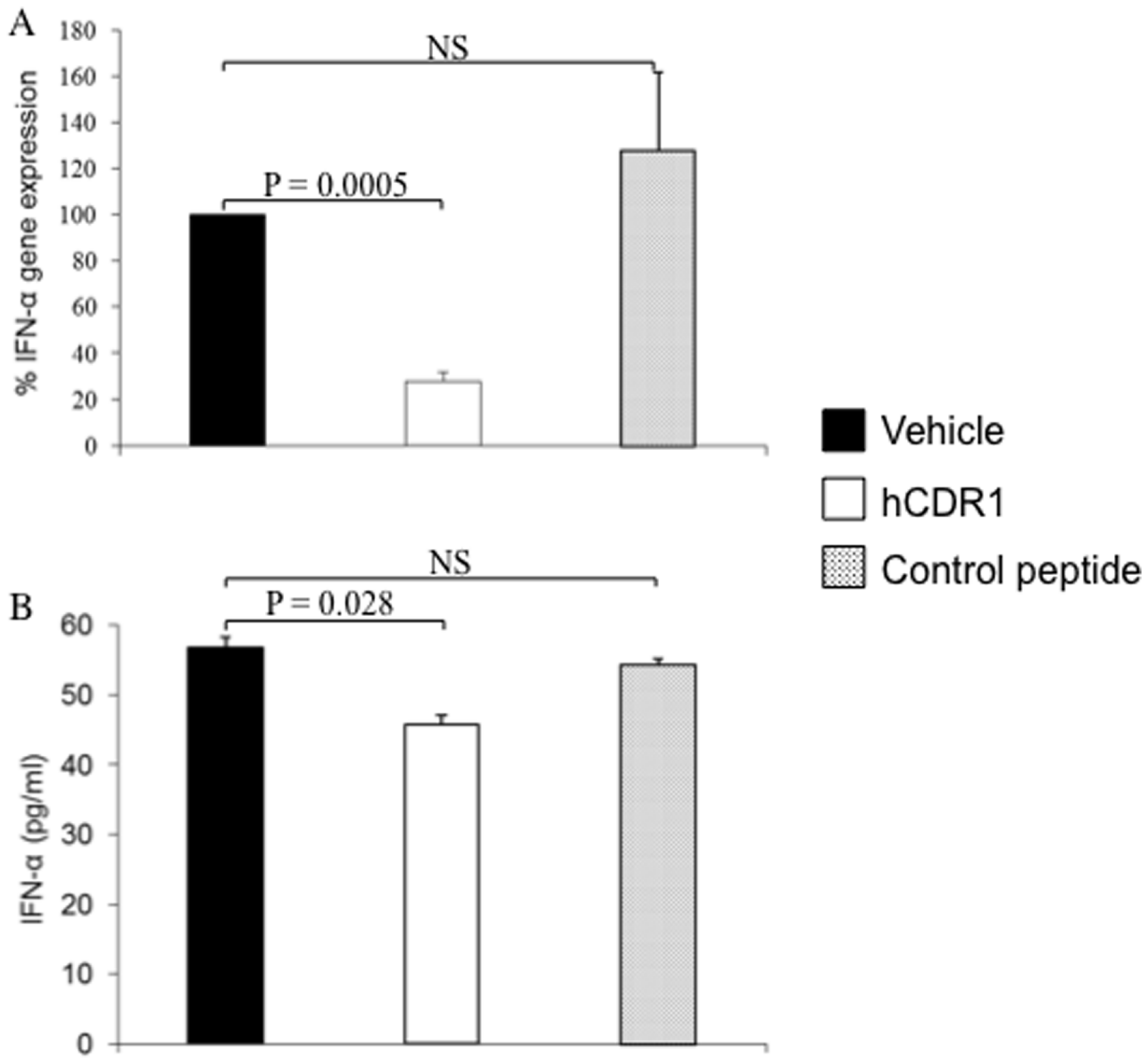
Treatment of SLE afflicted (NZBxNZW)F1 mice with hCDR1 results in the down regulation of IFN-α. (A) Mean percentage (±SE) results of 4 independent experiments in which the mRNA expression of IFN-α was determined in pools of spleen derived cells of SLE afflicted mice (10–12 mice per group) treated with vehicle, hCDR1, or the control, scrambled, peptide. The levels of gene expression were determined by real-time RT-PCR and calculated relatively to levels in cells from vehicle-treated mice (considered as 100%). (B) Mean concentrations (±SE) of IFN-α determined by ELISA in sera of the same groups of mice.

Figure 1A shows the effect of treatment with hCDR1 on IFN-α gene expression. The results are of 4 independent experiments in which mRNA was prepared from pools of spleen derived lymphocytes of SLE-afflicted mice treated with vehicle, hCDR1 or control peptide. The gene expression of IFN-α was determined by real-time RT-PCR. The results are expressed as percentage gene expression relatively to that observed for vehicle treated mice, considered as 100%. As can be seen in the Figure, treatment with hCDR1 down-regulated significantly (p = 0.0005) the gene expression of IFN-α as compared to levels of the vehicle treated mice. No such effects were observed in splenocytes of mice treated with the control peptide. In spite of the limited sensitivity of the ELISA assay, the levels of IFN-α (56.83±1.4 pg/ml) detected in sera of vehicle treated mice were similar to those reported for the sera of SLE patients [32]. As can been seen in Figure 1B, treatment with hCDR1 but not with the control peptide decreased significantly (p = 0.028) the sera levels of IFN-α. Thus, our studies demonstrated that hCDR1 treatment decreased IFN-α gene expression (Figure 1A) and its sera levels (Figure 1B).

In agreement, treatment with hCDR1 ameliorated disease manifestations in the experimental mice. As can be seen in Table 1, that summarizes the beneficial effects of hCDR1 treatment, a significant reduction was determined in the levels of dsDNA autoantibodies, in proteinuria levels as well as in glomerular immune complex deposits (ICD) as compared to mice that were treated with the vehicle alone. It is also seen in the Table that no such effects were observed in mice that were treated with the control peptide.

**Table 1:** Effects of treatment with hCDR1 on SLE manifestations in mice.

Treatment a	dsDNA Ab b(OD)	*p* c	Proteinuria d (g/L)	*p* c	ICD e	*p* c
Vehicle	0.50±0.08	—	8.36±1.86	—	2.43±0.2	—
hCDR1	0.30±0.05	0.04	1.94±0.45	0.03	1.57±0.2	0.002
Control^f^	0.52±0.08	NS	8.94±1.86	NS	2.78±0.2	NS

a SLE-afflicted (NZB×NZW)F1 mice (10–12 mice per group in 4 independent experiments) were treated with weekly subcutaneous injections of the vehicle, hCDR1, or a control (scrambled peptide) for 10 weeks.

b Results are of sera from mice that were bled after the end of treatment. Dilution of sera 1∶1250.

c Statistical evaluation was based on the Mann-Whitney U test to compare post –treatment effects between the vehicle –treated groups and the remaining treatment groups.

d Proteinuria was always measured at about the same time of day and all mice in an experimental cohort were tested together.

e Immune complex deposits (ICD) were assessed at sacrifice.

f p = 0.04, 0.02 and 0.0001 between the control peptide and hCDR1 treated mice for dsDNA specific antibodies, proteinuria and ICD, respectively.

**Figure 2:**
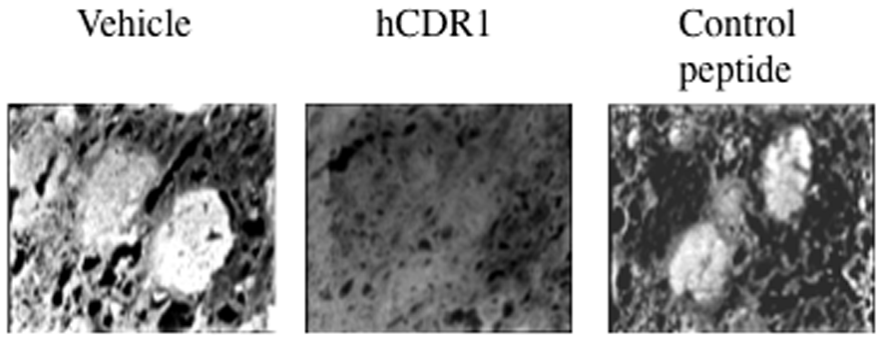
hCDR1 down regulates immune complex deposits in kidney sections. Immunohistology of kidney sections of representative mice of each experimental group (vehicle, hCDR1 and control peptide treated mice). Magnification X400.

Immunohistology of kidney sections of representative mice of each group are shown in Figure 2. As can been seen in the Figure, intense ICD are demonstrated in the kidney sections of the vehicle and the control peptide treated mice but not in the hCDR1 treated group of mice.

### Effects of hCDR1 on IFN-α gene expression of PBMC of lupus patients

**Figure 3:**
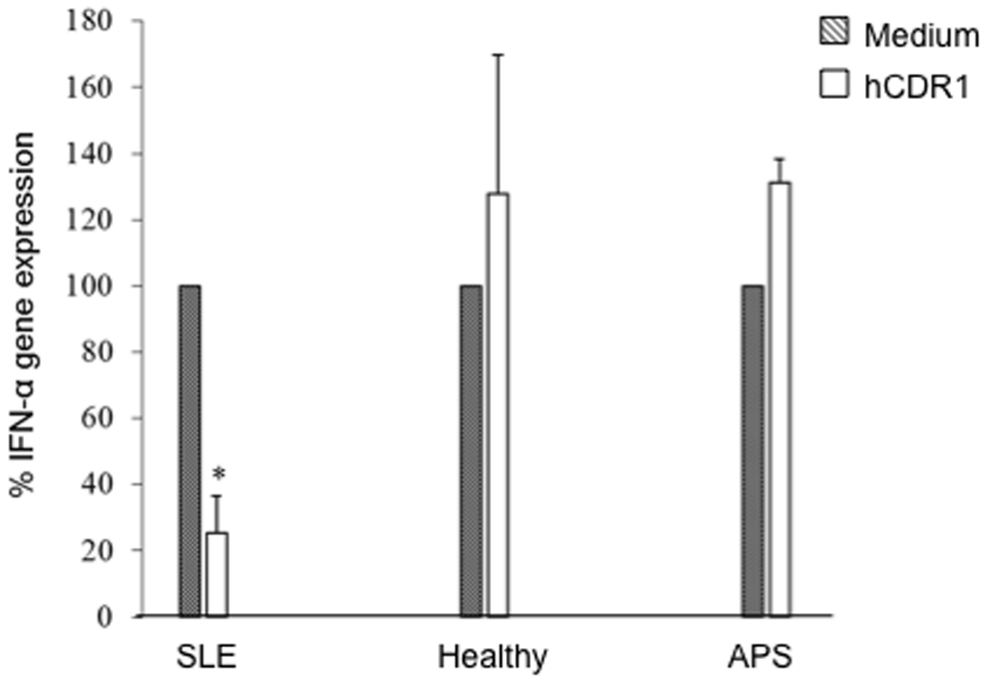
hCDR1 down regulates IFN-α gene expression in PBMC of SLE patients. PBMC of 10 SLE, 5 primary APS patients and 5 healthy controls were cultured (5×10^6^ cells/well) for 48 hours in the presence of medium or hCDR1 (25 µg/ml). Gene expression was determined by real-time RT-PCR. Results are presented as the mean±SE percentage of gene expression compared with cultures with medium (considered as 100%). * p = 0.004.

We further studied the in vitro effects of hCDR1 on IFN-α in lupus patients. To this end, PBMC were obtained from 10 lupus patients (8 females, 2 males) aged 32–65 years that were diagnosed with SLE according to four or more ACR diagnostic criteria [33]. Their main current clinical manifestations were arthritis (60%), mucocutaneous (50%), renal (20%) and pleuritis/pericarditis (20%). Eight patients were treated with Plaquonil (400 mg/d) and five with corticosteroids (2.5–10 mg/d of Prednisone) at the time of the study. The patients' PBMC were cultured for 4**8** hours in the presence of hCDR1 (25 µg/ml) or medium alone. Thereafter, mRNA was isolated from the cells and IFN-α gene expression was determined by real-time RT-PCR. For control we cultured concomitantly PBMC that were obtained from 5 healthy volunteers and PBMC of 5 patients with primary anti-phospholipid syndrome (APS) that did not have lupus (either clinically or serologically). Figure 3 shows the results of these experiments. It can be seen that in vitro incubation of PBMC of lupus patients with hCDR1 diminished significantly (p = 0.004) the IFN-α gene expression as compared with PBMC of the same patients cultured with medium (considered as 100%). It should be noted that the baseline levels of IFN-α gene expression in PBMC of SLE patients were 3 folds higher than those determined for the healthy controls (p = 0.028). Figure 3 also shows that in vitro incubation of hCDR1 with PBMC of healthy donors or of patients with primary APS did not down regulate the levels of IFN-α gene expression in the treated cells. Thus, these results suggest that the hCDR1-induced reduction of IFN-α gene expression is specific to lupus patients.

We also tested the supernatants of the lupus PBMC cultured with hCDR1 (or medium alone) for the presence of IFN-α by ELISA but apparently the assay was not sensitive enough and IFN-α could not be detected in the cultures.

### The effect of in vivo treatment of lupus patients with hCDR1 on IFN-α gene expression

**Figure 4:**
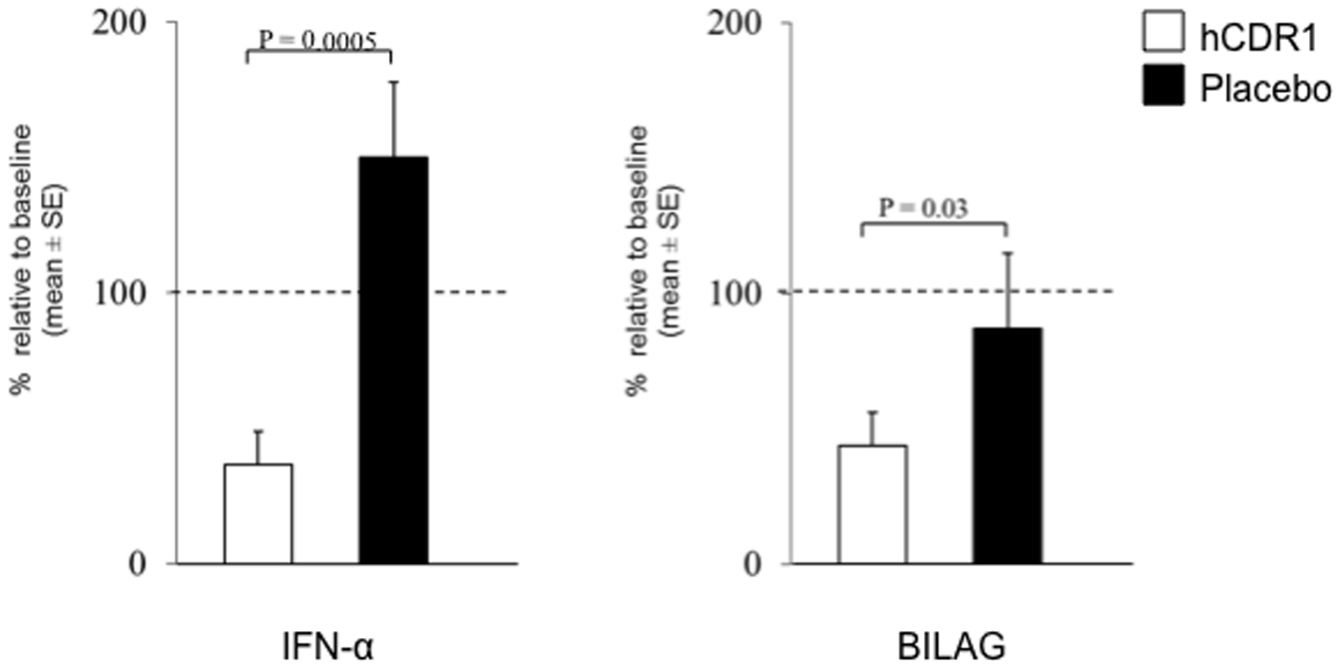
hCDR1 down regulates in vivo IFN-α gene expression in SLE patients. SLE patients were treated (subcutaneously, once a week) with either hCDR1 (0.5, 1, or 2.5 mg) or placebo. Gene expression in blood samples obtained from the patients was determined by real-time RT-PCR. Results are presented as mean percentage of gene expression (±SE) at week 24 compared to the levels at week 0 (defined as 100%; dotted line). Also shown in the Figure is the mean percent reduction in the BILAG score following 24 weeks of treatment with either hCDR1 or placebo as compared to the baseline score (week 0) considered as 100% (dotted line).

We have further evaluated the effect of hCDR1 treatment on IFN-α gene expression in 9 lupus patients with mild to moderate disease. The patients were treated with weekly subcutaneous injections of either hCDR1 (5 patients) or vehicle alone (4 patients). The inclusion and exclusion criteria, patients' characterization and clinical outcome were described previously [24]. Blood samples were collected from the patients in PAXgene tubes prior and following 24 weeks of treatment for the preparation of mRNA. The in vivo effect of weekly administration of hCDR1 to the SLE patients on IFN-α gene expression was then determined. Table 2 shows the expression of the IFN-α gene for the individual patients and Figure 4 presents the mean percent of gene expression at week 24 compared to the level prior to hCDR1 or vehicle treatment, at week 0 (defined as 100%; dotted line). As can be seen in Table 2 and Figure 4, treatment for 24 weeks with hCDR1 diminished significantly (p = 0.0005) the gene expression of IFN-α in the 5 treated patients. No significant effects were observed in the 4 lupus patients that were treated with the vehicle alone (placebo group; Table 2, Figure 4).

**Table 2:** The effect of in vivo treatment with hCDR1 on IFN-α gene expression in PBMC of SLE patients.

Patient No.	Treatment	Dose (mg)	IFN-α (% Expression relative to baseline)
70103	hCDR1	0.5	20
70106	hCDR1	0.5	20
70101	hCDR1	1.0	17
70403	hCDR1	1.0	44
70104	hCDR1	2.5	82
70404	Placebo	—	135
70405	Placebo	—	141
70102	Placebo	—	228
70107	Placebo	—	96

SLE patients with mild and moderate disease manifestations were treated (subcutaneously) with either hCDR1 or placebo. IFN-α gene expression in blood samples was determined by real-time RT-PCR. Results are presented as the percentage of gene expression at week 24 compared to that at week 0 (before the study was initiated), defined as100%.

Figure 4 also shows that the significant inhibition of IFN-α gene expression following treatment with hCDR1 was in agreement with the observed clinical effects. Thus, as shown in the Figure, lupus disease activity as determined by the BILAG score decreased significantly (p = 0.03) in the hCDR1 treated but not in the placebo treated group of patients. Not shown in the Figure are the SLEDAI scores of the treated patients. In agreement, a significant decrease in the SLEDAI-2K score (45% reduction, p = 0.02) was observed in the hCDR1 treated patients but not in the placebo treated patients [24].

### The role of IFN-α in the hCDR1-induced immunomodulation in SLE

**Figure 5:**
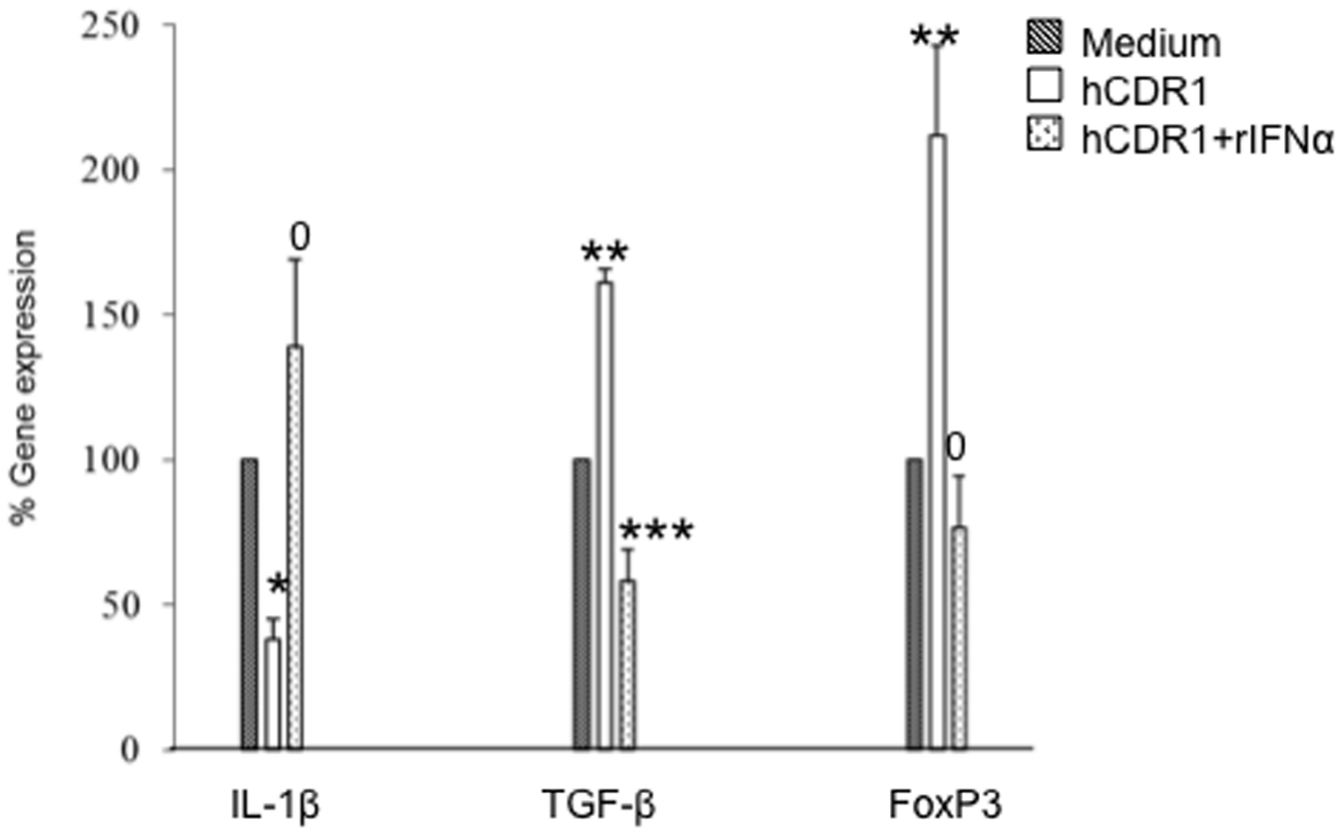
IFN-α diminishes hCDR1 immunomodulatory effects on PBMC of SLE patients. PBMC of 3 SLE patients were cultured (5×10^6^ cells/well) for 48 hours in the presence of medium, hCDR1 (25 µg/ml) or hCDR1 (25 µg/ml) and human recombinant IFN-α (rIFN-α) at a concentration of 5,000 U/ml. Gene expression (for IL-1β, TGFβ and FoxP3) were determined by real-time RT-PCR. Results are presented as the mean±SEpercentage of gene expression compared with cultures of PBMC incubated with medium alone (considered as 100%). *p = 0.05, **p = 0.03, ***p = 0.015 and ^0^ = not significant.

We further studied the possible mechanistic role of IFN-α in the hCDR1-induced immunomodulation in SLE. To this end, PBMC obtained from 3 SLE patients were cultured in triplicates for 48 hours in the presence of medium alone, hCDR1 (25 µg/ml) or hCDR1 (25 µg/ml) with human recombinant IFN-α at concentrations of 100–10,000 U/ml. Thereafter, mRNA was isolated from the cells and IL-1β, TGFβ and FoxP3 gene expression was determined by real-time RT-PCR. Figure 5 shows the results of these experiments using human recombinant IFN-α at a concentration of 5,000 U/ml (shown to have the optimal effect). It can been seen that, as was previously shown [23], hCDR1 significantly down-regulated IL-1β and up-regulated TGFβ and FoxP3 gene expression in lupus PBMC (p = 0.05, 0.03 and 0.03 for IL-1β, TGFβ and FoxP3, respectively). The addition of IFN-α to the cultures abolished completely those effects (p = 0.05, 0.016 and 0.028 between cultures of PBMC with hCDR1 and those with hCDR1+recombinant IFN-α for IL-1β, TGFβ and FoxP3, respectively) suggesting that IFN-α plays a role in the immunomodulating effects of hCDR1 in SLE.

## Discussion

The main finding of the present study is that the tolerogenic peptide hCDR1 is capable of suppressing IFN-α gene expression in a murine SLE model and in lupus patients. This suppressive effect was specific since it was not observed following treatment of (NZBxNZW) F1 lupus prone mice with the control scramble peptide, or in PBMC obtained from healthy volunteers or APS patients. Moreover, the down regulation of IFN-α gene expression correlates to the therapeutic beneficial effects of hCDR1 in murine and in human lupus.

In previous studies, we were able to demonstrate that the tolerogenic peptide, hCDR1, ameliorates manifestations of SLE in murine models [9] and in a small cohort of lupus patients [24]. Those beneficial effects resulted from the effects of hCDR1 on different immune cell types (including dendritic [22], T [9], [17], [34] and B cells [20], [21], [35]) and on cytokines [9], [11]. Thus, hCDR1 down regulated (in vivo and in vitro, in murine SLE models and in human lupus) pro-inflammatory cytokines [11], [23], [24], apoptotic factors [18], [19], [23], [24], [36], B-cell stimulatory factors (BAFF/BLyS) [20], [24] and up regulated immunosuppressive cytokines [9], [11], [23], [24], [37] with the induction of CD4+CD25+FoxP3+regulatory T-cells [15], [23].

We demonstrate here a significant down regulation of IFN-α gene expression by hCDR1 in three different lupus related experimental systems. First, treatment of (NZBxNZW) F1 lupus prone mice with hCDR1, but not with the vehicle or the control (scrambled) peptide, resulted in significant down regulation of IFN-α gene expression (Figure 1A) and IFN-α sera levels (Figure 1B) which correlated to the serological and clinical beneficial effects of hCDR1 in this model (Table 1, Figure 2). Second, hCDR1 significantly decreased, in vitro, IFN-α gene expression in PBMC of lupus patients but not in PBMC obtained from healthy volunteers or primary APS patients (Figure 3). Thus, as was previously shown for other cytokines and immunosuppressive factors [9], [23], the effect of hCDR1 on IFN-α is specific to lupus. Third, treatment of five lupus patients with hCDR1 for twenty-four consecutive weeks resulted in significant inhibition of IFN-α gene expression (Table 2, Figure 4). Concomitantly, disease activity (defined by both, BILAG and SLEDAI) in the hCDR1 treated patients decreased significantly (Figure 4 and [24]). No such effects were observed in the four other lupus patients who were treated with the vehicle alone [Table 2, Figure 4]. Taken together, the present studies clearly demonstrated lupus-specific inhibitory effects of our tolerogenic peptide on IFN-α in SLE.

We have previously demonstrated the ability of the tolerogenic peptide, hCDR1, to ameliorate SLE manifestations by immunomodulating specifically a variety of cytokines, molecules and cell types that are involved in the pathogenicity of lupus. In the present study we demonstrated down regulating effect of hCDR1 on one of the important cytokines that is involved in lupus etiology and pathogenesis, namely IFN-α. Recent reports showing that IFN-α has the potential to influence the development and progression of SLE suggest this cytokine as a therapeutic target. A number of mechanisms were suggested to account for the pathogenic effects of IFN-α. It has been reported that dendritic cells mature and become more prone to activate T cells in the presence of IFN-α [38]. Further, activity of regulatory T cells (Tregs) was shown to be suppressed by the in vitro treatment of dendritic cells with IFN-α [39] and the increased levels of IFN-α in lupus patients was reported to contribute, at least in part, to the diminished Tregs activity observed in patients with SLE [40]. Type I interferons were shown to directly improve B-cell survival in vitro [41] and to reduce the sensitivity of B cells to FasL-mediated apoptosis [42]. In addition, IFN-α may affect B cell survival, maturation and differentiation, indirectly by inducing dendritic cells and macrophages to produce BLyS [43]. Moreover, the present study suggests that IFN-α plays a mechanistic role in the immunomodulating effects of hCDR1 in SLE since the addition of recombinant IFN-α diminished the effects of hCDR1 on cytokine expression in PBMC of SLE patients (Figure 5). In agreement, the addition of anti-IFN-α-antibodies to PBMC of SLE patients (in the absence of hCDR1) down regulated IL-1β and up regulated TGFβ and FoxP3 gene expression, similarly to the immunomodulation of these genes by hCDR1 (Mozes et al., unpublished results) further supporting the role of IFN-α.

Thus, the diminished expression of IFN-α following treatment with hCDR1 demonstrated in our study may affect SLE manifestations via any or all the above suggested mechanisms. Indeed, we have previously reported that treatment with hCDR1 down regulated the maturation and activation of dendritic cells [22] resulting in the induction of functional Tregs and suppressed autoreactive T cell activity in SLE models and in lupus patients [9]. Furthermore, hCDR1 was shown to reduce BAFF/BLyS production and to up-regulate B cell apoptosis via the up regulation of pro-apoptotic molecules (e.g. Caspase 8) and the down regulation of anti-apoptotic molecules (Bcl-2, Bcl-xL) [20], [24]. Nevertheless, it should be kept in mind that even though the effects of IFN-α can explain many SLE features, only a fraction of SLE patients displays elevated levels of IFN-α. Furthermore, other pathogenic cytokines that function together with IFN-α or independently, and cell marker molecules [2], [44] were shown to be involved in lupus and therefore, reducing IFN-α probably affects only partially this complex, multifactorial disease. Thus, blocking a single cytokine might not be the full answer for controlling SLE. Indeed, we have previously shown that treatment with hCDR1 leads to a cascade of events that affect activated dendritic cells, T and B cells and their products as well as important pathways involved in the pathogenesis of lupus [9], [45], [46].

An important aim in the treatment of lupus, as well as any other diseases, is to suppress the SLE related autoimmune responses and, at the same time, to spare the normal function of the immune system. One of the challenges using therapeutic means that neutralize IFN-α is to inhibit the SLE-related over production of IFN-α and to leave intact the anti-viral activity of IFN-α. A similar problem arises when certain cell types (e.g., B cells) are depleted in order to suppress autoimmune responses. We have shown, in the present study that the inhibitory effect of hCDR1 on the expression of the IFN-α gene is specific to lupus and it does not affect healthy controls and patients with APS (Figure 3). In agreement, we have previously shown that hCDR1 inhibited in vitro murine and human T cell proliferation as well as IFN-γ and IL-2 production only in cases of lupus associated responses [14], [34], [37], [45] and did not affect responses to unrelated antigens. Similarly, we demonstrated that treatment of SLE–like disease in SCID mice, transplanted with PBMC of SLE patients, led to the suppressed production of the human anti-dsDNA autoantibodies as well as to the amelioration of SLE manifestations. Nevertheless, no significant effects could be observed on the levels of human anti-tetanus toxoid antibodies [47] in the treated mice. Moreover, only Tregs induced by hCDR1 ameliorated disease manifestations when transferred into SLE afflicted (NZBxNZW)F1 mice [15]. Tregs originating from a control peptide or the vehicle treated mice did not have a significant clinical effect on mice with established lupus [15]. The specific effect of hCDR1 was further confirmed by the fact that hCDR1 induced functional Tregs were not capable of inhibiting myasthenia gravis associated responses (Mozes E. et al. unpublished results).

Thus, the efficient and specific beneficial effects of hCDR1 at the different checkpoints and on the various factors involved in lupus, as exemplified in the present study by its effect on one of the central cytokine, IFN-α, suggest a potential role for this tolerogenic peptide in the treatment of lupus patients.

## Materials and Methods

### Mice

Female (NZBxNZW)F1 mice were purchased from The Jackson Laboratory (Bar harbor, ME, USA). Murine experiments were approved by the Animal Care and Use Committee of the Weizmann Institute of Science.

### Synthetic peptides

A peptide, GYYWSWIRQPPGKGEEWIG, (hCDR1) [37] that is based on the complementarity determining region (CDR) 1 of the human anti-DNA monoclonal antibody, bearing a major idiotype (16/6 Id) [10] was synthesized by Polypeptide Laboratories (CA, USA). A peptide, SKGIPQYGGWEGWRYEI, containing the same amino acids as hCDR1 in a scrambled order was used as a control.

### Treatment of (NZBxNZW)F1 mice

Eight-month old female mice (10–12 mice per group) with established lupus manifestations were treated in 4 independent experiments with 10 weekly subcutaneous injections of hCDR1 (50 µg/mouse), control peptide (50 µg/mouse) or vehicle alone (phosphate buffered saline).

### Evaluation of murine lupus disease activity

Anti-dsDNA autoantibody levels were measured using λ phage dsDNA, as previously described [11]. Proteinuria was measured by a standard semi-quantitative test, using an Albustix kit (Bayer Diagnostic, Newbury, UK). Detection of glomerular immune complex deposits was performed as described earlier [11]. The intensity of immune complex deposits (immunohistology) was graded as follows: 0, no immune complex deposits; 1, low intensity; 2, moderate intensity; and 3, high intensity of immune complexes. The analysis was performed by two people blinded to whether the mice belonged to control or experimental groups.

### Patients

Ten lupus patients (8 females and 2 males), 5 patients (all females) with primary APS and 5 (4 females and 1 male) age matched healthy controls participated in the in vitro experiments. We also present here data of 9 lupus patients (8 females and 1 male) from two Israeli Medical Centers. These patients participated in a large clinical trial with hCDR1 (Edratide) [24]. Included are all patients from the two Medical Centers who completed the study and from whom blood samples were taken at least twice (before treatment initiation and at week 24) for mRNA preparation. All lupus patients were diagnosed according to the American College of Rheumatology (ACR) diagnostic criteria [33]. All participants signed an informed consent form prior to the initiation of the studies. The studies were approved by the Ethic Committees of the Medical Centers and were conducted according to all good clinical practice (GCP) rules.

### In vitro experiments

Peripheral blood mononuclear cells (PBMC) were isolated from heparinized venous blood using UNI-SEP maxi for density gradient separation (NOVAmed Ltd., Jerusalem, Israel). PBMC (5×10^6^/ml) were cultured in triplicates in enriched RPMI-1640 medium containing 10% fetal calf serum [23] for 48 hours in the presence of hCDR1 (25 µg/ml) or medium alone as control. In some experiments, PBMC were also cultured with hCDR1 (25 µg/ml) and various concentrations (100–10,000 U/ml) of human recombinant IFN-α (Millipore, Temacula, Ca, USA). PBMC were then washed (x3 in RPMI-1640) and mRNA was extracted for gene expression as described below.

### In vivo studies

The 9 lupus patients that participated in the clinical study had a mild to moderate disease with SLE-disease activity index 2000 (SLEDAI-2K) [48] of 6–12 (inclusive) and stable lupus-related medications [24]. hCDR1 dissolved in Captisol (Sulfobutyl ether cyclodextrin sodium, CyDex, Inc., KS, USA) was injected subcutaneously weekly for 24 consecutive weeks at doses of 0.5 mg (2 patients), 1 mg (2 patients) or 2.5 mg (1 patient). Four patients were treated with Captisol alone. Patients were evaluated clinically by the SLEDAI-2K and the British Islets Lupus Assessment Group (BILAG) [49] scores. Venous blood samples prior (week 0) and following treatment (week 24) were collected in PAXgene (PreanalytiX, Switzerland) tubes and frozen at -70°C until mRNA isolation.

### Real-time RT-PCR

Total RNA was isolated from spleen derived murine lymphocytes, human PBMC or blood samples collected in PAXgene tubes. The RNA was reversed transcribed to prepare cDNA using Moloney murine leukemia virus reverse transcribtase (Promega, Madison, WI, USA). The resulting cDNA was subjected to real-time RT-PCR using Light Cycler ((Roche Mannheim, Germany) according to the manufecturer's instructions. Primer sequences (forward and reversed, respectively) were: mouse IFN-α1 (5′-CTGCAAGGCTGTCTGA-3′, 5′-GCACATTGGCAGAGGA-3′), mouse β-actin, (5′-GTGACGTTGACATCCG-3′, 5′-CAGTAACAGTCCGCCT-3′), human IFNα1 (5′-TGTGATCTCCCTGAGACC-3′, 5′-AGATGGAGTCCGCATT-3′), human IL-1β (5'-CAGAAAACATGCCCGT-3', 5'-GCACTACCCTAAGGCAG-3'), human TGF-β (5'-GCAAGACTATCGACATGG-3', 5'-ACTTGTCATAGATTTCGTTGTG-3'), human FoxP3 (5'-CCACAACATGGACTACTT-3', 5'-CGTTTCTTGCGGAACT-3'), and human GAPDH (5′-CTGCCAACGTGTCAGT-3′, 5′- GTTGAGGGCAATGCCA-3′). The levels of β-actin (murine studies) and GAPDA (human studies) were used to normalize the gene expression levels of the other genes.

### ELISA assays for IFN-α

IFN-α levels in murine sera and in human PBMC supernatants were determined by Platinum ELISA sets (eBioscience, San Diego, Ca.) according to the manufacturer's instructions.

### Statistical analysis

Results are presented as Mean±standard error (SE). The nonparametric Mann-Whitney and unpaired Student's T tests were used for statistical analysis. *p* values of 0.05 or less were considered statistically significant.
